# Acknowledgment of Reviewers

**DOI:** 10.2188/jea.JE20230308

**Published:** 2023-12-05

The following scientists assisted the journal by reviewing manuscripts during the period November 1, 2022 to October 31, 2023.

We gratefully acknowledge their critical evaluation and valuable assistance in selecting articles for publication.

**Table:** 

Abd El-Rahim, Ibrahim
Abe, Sarah K.
Aizawa, Yuta
Amagasa, Shiho
Ando, Yuichi
Anzai, Tatsuhiko
Arashiro, Takeshi
Chen, Jie
Choi, Ji-Yeob
Cooray, Upul
Dimeglio, Chloé
Ekuni, Daisuke
Fujii, Takako
Fujiwara, Aya
Fujiyoshi, Akira
Fukui, Keisuke
Fukushima, Noritoshi
Fukushima, Wakaba
Gallus, Silvano
Goto, Tadahiro
Hagiwara, Yasuhiro
Hamaguchi, Masahide
Harada, Sei
Hayashi, Katsuma
Hirayama, Atsushi
Hirokawa, Kumi
Hisamatsu, Takashi
Hishida, Asahi
Honyashiki, Mina
Horita, Nobuyuki
Hosozawa, Mariko
Ichikawa, Shuhei
Igeta, Masataka
Iida, Miho
Inoue, Kosuke
Inoue, Norihiko
Itani, Osamu
Iwagami, Masao
Iwasa, Hajime
Iwasaki, Motoki
Jiang, Guangming
Jindai, Kazuaki
Jinnouchi, Hiroshige
Kabasawa, Keiko
Kadota, Aya
Kajiwara Saito, Mari
Kakizaki, Masako
Kamigaki, Taro
Kashima, Saori
Kashino, Ikuko
Kasuga, Yoshifumi
Kataoka, Yuki
Katsuragawa, Sho
Katsuura-Kamano, Sakurako
Kawakami, Ryoko
Kim, Jeongseon
Kim, Kyeezu
Kishimoto, Hiro
Kitamura, Tetsuhisa
Kiyohara, Kosuke
Ko, Yura
Kobayashi, Sumitaka
Kondo, Takaaki
Koyanagi, Yuriko
Kuriki, Kiyonori
Kuroda, Yujiro
Kurokawa, Naoyuki
Kusama, Taro
Kusunoki, Takashi
Kutsuna, Satoshi
Kuwabara, Masanari
Kuwahara, Keisuke
Lee, Dong-Wook
Li, Jiaqi
Lin, Nan
Lin, Yingsong
Lugo, Alessandra
Ma, Defu
Machida, Munehito
Maeda, Eri
Masago, Yoshifumi
Matsuyama, Yusuke
Mawatari, Momoko
Metoki, Hirohito
Michikawa, Takehiro
Min, Kyoung-Bok
Miyake, Yoshihiro
Miyamoto, Yoshihisa
Miyata, Jun
Mizoue, Tetsuya
Momma, Haruki
Morimoto, Libby
Morita, Ichizo
Murakami, Keiko
Muraki, Isao
Nakajima, Atsushi
Nakajima, Mikio
Nakamoto, Mariko
Nakano, Shiori
Nakashima, Kei
Nawa, Nobutoshi
Nexø, Mette
Nishioka, Norihiro
Nishiyama, Takeshi
Nojiri, Shuko
Oba, Mari S.
Obara, Taku
Ochi, Manami
Ohfuji, Satoko
Ohnishi, Hirofumi
Ojima, Toshiyuki
Okada, Yohei
Onishi, Kazunari
Ono, Rei
Onozuka, Daisuke
Orihara, Shunichiro
Osaki, Yoneatsu
Osawa, Itsuki
Otsuka, Yuichiro
Oze, Isao
Park, Boyoung
Possenti, Irene
Qin, Li-Qiang
Rossi, Marta
Saika, Kumiko
Saito, Eiko
Sako, Akahito
Sakurai, Masaru
Sando, Eiichiro
Sato, Shuntaro
Savitz, David
Sawaya, Yohei
Scala, Marco
Seino, Satoshi
Shimada, Ryo
Shimokata, Hiroshi
Shin, Jung-ho
Shinjoh, Masayoshi
Shinozaki, Tomohiro
Suah, Jing Lian
Sugawara, Yuka
Sugimoto, Minami
Sugiura, Tomoko
Sugiyama, Hiromi
Suma, Shino
Suzuki, Etsuji
Suzuki, Harumitsu
Suzuki, Yuka
Tabuchi, Takahiro
Tachimori, Hisateru
Tajima, Takayuki
Takakura, Minoru
Takebayashi, Yoshitake
Takeuchi, Jiro
Takeyama, Makoto
Tam, Cherry
Tanaka, Hideo
Tang, Julian
Tani, Yukako
Terui, Toshihiro
Tian, Haodong
Tigova, Olena
Tsuchiya, Naho
Tsuda, Toshihide
Tsuji, Taishi
Ueda, Yutaka
Ueta, Kayo
Ukawa, Shigekazu
Umesawa, Mitsumasa
Uno, Shunsuke
Utsunomiya, Takeshi
Wakai, Kenji
Wang, Hualin
Weng, Lu-Chen
Yachi, Sen
Yamakawa, Michiyo
Yamamoto, Kei
Yamana, Hayato
Yasui, Toshiyuki
Yatsuya, Hiroshi
Yoh, Tomoaki
Yokoyama, Yuri
Yoshida, Satomi
Yoshimoto, Takahiko
Zaitsu, Masayoshi

